# Leveraging menopause to uncover novel insights into Alzheimer's disease pathophysiology

**DOI:** 10.1002/alz70856_100377

**Published:** 2025-12-24

**Authors:** Kaitlin B Casaletto, Rowan Saloner

**Affiliations:** ^1^ Memory and Aging Center, UCSF Weill Institute for Neurosciences, University of California, San Francisco, San Francisco, CA, USA; ^2^ University of California San Francisco, San Francisco, CA, USA

## Abstract

**Background:**

Females are at two‐fold increased risk of Alzheimer's disease (AD). Leveraging naturally‐occurring female biology may provide new insights into AD pathophysiology and address knowledge gaps underlying female‐predominant risk. Younger age of menopause is associated with AD risk, especially tau pathology later in life. We examined plasma proteomics to identify biological targets linking menopause timing to tau.

**Method:**

Functionally‐intact postmenopausal women (*n* = 77) and men (*n* = 71) had plasma analyzed for 132 proteins via ultra‐sensitive NUcleic acid Linked Immuno‐Sandwich Assay. Protein levels were adjusted for age. Bivariate correlations screened associations between age at menopause and protein levels in females (bivariate *p*‐value<0.05). Given age at menopause associated with multiple tau biomarkers, we identified proteins correlated with both menopause age and tau biomarkers at r≥0.20 and tested whether these proteins statistically mediated relationships between age of menopause and tau biomarkers. Candidate targets were then evaluated for sex‐specificity in associations with tau across females and males.

**Result:**

Seventeen proteins associated with menopause age. Older age at menopause associated with lower tau (ptau181, ptau231, MAPT), immune/inflammatory (e.g., TREM2, IL6, C1QA, IFNy, TNF), and metabolic (MDH1), and higher synaptic markers (e.g., NPTX2, BDNF, SNCA). Given high representation of tau biology, we evaluated proteomic overlap between menopause age and the identified tau markers (ptau181, ptau231, and MAPT). 11 proteins correlated with both menopause age and all three tau proteins. Of the 11 candidates, only AGRN statistically mediated the relationship between age of menopause and each tau measure (38‐47% variance explained); however, all 11 factors showed significant associations with ptau181, ptau231, and MAPT in both males and females suggesting relevance to tau biology broadly. Of the 11 factors, AGRN, IL10, IL2, and CCL3 showed sex‐specific interactions such that the positive association between each protein and tau biomarkers was disproportionately stronger in females versus males.

**Conclusion:**

Leveraging female‐specific biology can uncover new insights into tau biology. In postmenopausal women, age of menopause associated with a range of biological factors relevant for brain aging, particularly immune, synaptic, and tau biology. AGRN, a secreted extracellular matrix heparin‐sulfate glycoprotein, most consistently associated with menopause‐related tau biomarkers and may be a candidate to explain female‐specific vulnerability to tau.

